# Supplementary material for: Impact of measured versus estimated glomerular filtration rate-based screening on living kidney donor characteristics: A study of multiple cohorts
Source: PLoS One. 2022 Jul 7;17(7):e0270827. doi: 10.1371/journal.pone.0270827 (PMC9262218; doi:10.1371/journal.pone.0270827)
Supplement: S6 Fig — Differences between mean pre-donation eGFR were tested using the independent sample T-test, P-values are shown in the figure. Pre-donation mGFR in the mGFR-cohort was added on the right in the figure. (DOCX) [file pone.0270827.s006.docx]

| 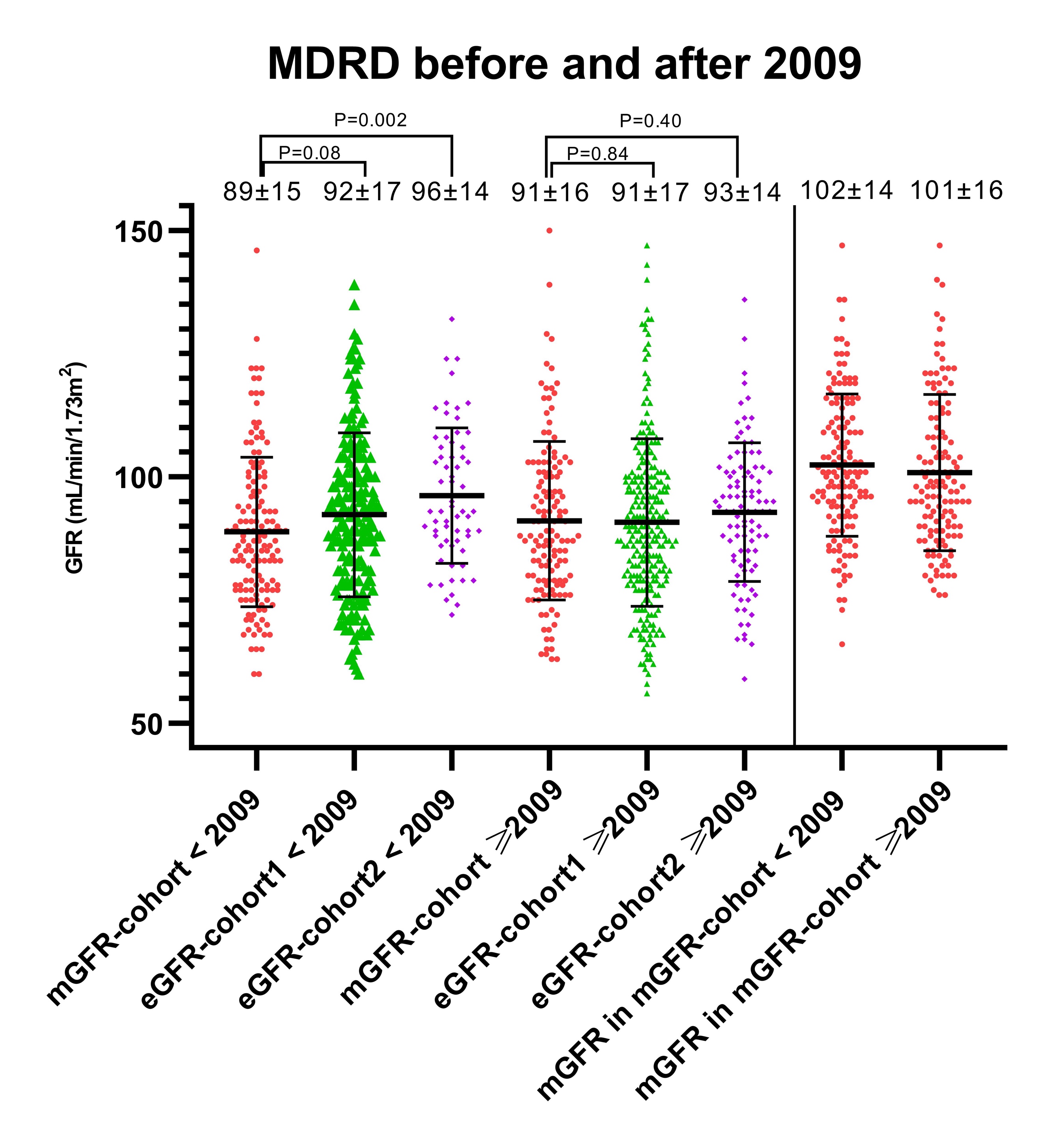 |
| --- |
| **Figure S6. Distribution of pre-donation eGFR (MDRD) before and after 2009 per center.** Differences between mean pre-donation eGFR were tested using the independent sample T-test, P-values are shown in the figure. Pre-donation mGFR in the mGFR-cohort was added on the right in the figure. |
