# Supplementary material for: Impact of measured versus estimated glomerular filtration rate-based screening on living kidney donor characteristics: A study of multiple cohorts
Source: PLoS One. 2022 Jul 7;17(7):e0270827. doi: 10.1371/journal.pone.0270827 (PMC9262218; doi:10.1371/journal.pone.0270827)
Supplement: S1 Table — Bias calculated as mGFR/BSA−eGFR. Abbreviations: eGFR: Estimated glomerular filtration rate; mGFR/BSA: Measured glomerular filtration rate corrected for BSA; BSA: Body surface area; IQR: Interquartile range. (DOCX) [file pone.0270827.s007.docx]

| **Table S1. Pre- and five year post-donation bias between eGFR and mGFR_/BSA_ in the mGFR-cohort** | | |
| --- | --- | --- |
|  | **Pre-donation** | **5 year post-donation** |
| Mean bias | -10 | -5 |
| Standard deviation | 12 | 9 |
| Median bias | -10 | -6 |
| IQR | -19 to -2 | -12 to 0 |
| Range | -48 to 19 | -34 to 20 |
| Bias calculated as eGFR – mGFR_/BSA_  Abbreviations: eGFR: estimated glomerular filtration rate; mGFR_/BSA_: measured glomerular filtration rate corrected for BSA; BSA: body surface area; IQR: interquartile range. | | |
