# Supplementary material for: Impact of measured versus estimated glomerular filtration rate-based screening on living kidney donor characteristics: A study of multiple cohorts
Source: PLoS One. 2022 Jul 7;17(7):e0270827. doi: 10.1371/journal.pone.0270827 (PMC9262218; doi:10.1371/journal.pone.0270827)
Supplement: S2 Table — Bias calculated as CrCl–mGFR/BSA. Abbreviations: CrCl: 24 hour creatinine clearance; mGFR/BSA: Measured glomerular filtration rate corrected for BSA; BSA: Body surface area; SD: Standard deviation; IQR: Interquartile range. (DOCX) [file pone.0270827.s008.docx]

| **Table S2. Pre- and post-donation bias between CrCl and mGFR_/BSA_ in the mGFR-cohort** | | |
| --- | --- | --- |
|  | *Pre-donation* | *5y post-donation* |
| Mean bias | 26 | 18 |
| SD | 29 | 19 |
| Median bias | 23 | 16 |
| IQR | 7 to 44 | 6 to 29 |
| Range | -50 to 128 | -39 to 73 |
| Bias calculated as CrCl – mGFR_/BSA_  Abbreviations: CrCl: 24 hour creatinine clearance; mGFR_/BSA_: measured glomerular filtration rate corrected for BSA; BSA: body surface area; SD: standard deviation; IQR: interquartile range. | | |
