# Supplementary material for: Impact of measured versus estimated glomerular filtration rate-based screening on living kidney donor characteristics: A study of multiple cohorts
Source: PLoS One. 2022 Jul 7;17(7):e0270827. doi: 10.1371/journal.pone.0270827 (PMC9262218; doi:10.1371/journal.pone.0270827)
Supplement: S3 Table — Binary variables presented as n (%), continuous variables presented as mean ±SD. Abbreviations: CKD-EPI: Chronic kidney disease epidemiology collaboration equation; CrCl: Creatinine clearance; mGFR: Measured GFR; BMI: Body mass index; BSA: Body surface area; SBP: Systolic blood pressure; DBP: Diastolic blood pressure; SD: Standard deviation. (DOCX) [file pone.0270827.s009.docx]

| **Table S3. Pre-donation characteristics of donors from the mGFR-cohort and eGFR-cohorteGFR-cohort2 with an underestimation of CrCl by eGFR *≥*10 mL/min.** | | | | | | |
| --- | --- | --- | --- | --- | --- | --- |
|  | **mGFR-cohort** | | | **eGFR-cohort2** | | |
|  | ***Underestimation ≥10 mL/min*** | ***Underestimation <10 mL/min*** | **P value** | ***Underestimation ≥10 mL/min*** | ***Underestimation <10 mL/min*** | **P value** |
| Number, N (%) | 188 | 36 | - | 139 | 21 | - |
| CKD-EPI, mL/min/1.73m^2^ | 90 ±13 | 93 ±11 | 0.21 | 94 ±12 | 95 ±10 | 0.63 |
| CrCl, mL/min | 135 ±30 | 86 ±15 | <0.001 | 135 ±25 | 94 ±14 | <0.001 |
| mGFR, mL/min | 117 ±22 | 102 ±16 | <0.001 | - | - | - |
| mGFR_/BSA_, mL/min/1.73m^2^ | 102 ±15 | 97 ±11 | 0.03 | - | - | - |
| Age, years | 52 ±9 | 56 ±12 | 0.06 | 53 ±10 | 55 ±7 | 0.37 |
| Female sex, n (%) | 93 (50) | 28 (78) | 0.002 | 55 (40) | 17 (81) | <0.001 |
| Caucasian race, n (%) | 188 (100) | 36 (100) | - | 139 (100) | 21 (100) | - |
| Weight, kg | 83 ±13 | 71 ±11 | <0.001 | 80 ±13 | 66 ±13 | <0.001 |
| Height, cm | 175 ±9 | 168 ±9 | <0.001 | 174 ±8 | 166 ±8 | <0.001 |
| BMI, kg/m^2^ | 27 ±3 | 25 ±4 | 0.01 | 26 ±3 | 24 ±4 | 0.002 |
| BSA, m^2^ | 1.98 ±0.19 | 1.80 ±0.15 | <0.001 | 1.94 ±0.18 | 1.73 ±0.17 | <0.001 |
| SBP, mmHg | 128 ±14 | 129 ±15 | 0.73 | 138 ±15 | 135 ±22 | 0.56 |
| DBP, mmHg | 77 ±9 | 77 ±8 | 0.83 | 81 ±9 | 79 ±8 | 0.21 |
| Serum creat, µmol/L | 76 ±13 | 68 ±11 | 0.001 | 74 ±12 | 65 ±7 | <0.001 |
| Binary variables presented as n (%), continuous variables presented as mean ±SD  Abbreviations: CKD-EPI: chronic kidney disease epidemiology collaboration equation; CrCl: creatinine clearance; mGFR: measured GFR; BMI: body mass index; BSA: body surface area; SBP: systolic blood pressure; DBP: diastolic blood pressure; SD: standard deviation. | | | | | | |
