# Supplementary material for: Impact of measured versus estimated glomerular filtration rate-based screening on living kidney donor characteristics: A study of multiple cohorts
Source: PLoS One. 2022 Jul 7;17(7):e0270827. doi: 10.1371/journal.pone.0270827 (PMC9262218; doi:10.1371/journal.pone.0270827)
Supplement: S4 Table — Binary variables presented as n (%), continuous variables presented as mean ±SD. Abbreviations: CKD-EPI: Chronic kidney disease epidemiology collaboration equation; CrCl: Creatinine clearance; mGFR: Measured GFR; BMI: Body mass index; BSA: Body surface area; SBP: Systolic blood pressure; DBP: Diastolic blood pressure; SD: Standard deviation. (DOCX) [file pone.0270827.s010.docx]

| **Table S4. Pre-donation characteristics of donors from the mGFR-cohort with an overestimation of mGFR_BSA_ by eGFR.** | |
| --- | --- |
| Number, N(%) | 45 |
| Overestimation | -6 ±5 |
| CKD-EPI, mL/min/1.73m^2^ | 98 ±10 |
| CrCl, mL/min | 122 ±28 |
| mGFR, mL/min | 105 ±18 |
| mGFR_/BSA_, mL/min/1.73m^2^ | 92 ±10 |
| Age, years | 53 ±10 |
| Female sex, n (%) | 27 (60) |
| Caucasian race, n (%) | 45 (100) |
| Weight, kg | 82 ±14 |
| Height, cm | 174 ±10 |
| BMI, kg/m^2^ | 27 ±4 |
| BSA, m^2^ | 1.97 ±0.20 |
| SBP, mmHg | 125 ±14 |
| DBP, mmHg | 76 ±9 |
| Serum creat, µmol/L | 67 ±12 |
| Binary variables presented as n (%), continuous variables presented as mean ±SD  Abbreviations: CKD-EPI: chronic kidney disease epidemiology collaboration equation; CrCl: creatinine clearance; mGFR: measured GFR; BMI: body mass index; BSA: body surface area; SBP: systolic blood pressure; DBP: diastolic blood pressure; SD: standard deviation. | |
