# Supplementary material for: Impact of measured versus estimated glomerular filtration rate-based screening on living kidney donor characteristics: A study of multiple cohorts
Source: PLoS One. 2022 Jul 7;17(7):e0270827. doi: 10.1371/journal.pone.0270827 (PMC9262218; doi:10.1371/journal.pone.0270827)
Supplement: S5 Table — Abbreviations: eGFR: Estimated glomerular filtration rate. (DOCX) [file pone.0270827.s011.docx]

| **Table S5. Number of donors with eGFR above and under age-adapted threshold according to the Dutch Living Kidney Donor Guidelines.** | | | | | | | |
| --- | --- | --- | --- | --- | --- | --- | --- |
|  |  | *mGFR-cohort* | | *eGFR-cohort1* | | *eGFR-cohort2* | |
| **Age category** | **eGFR threshold** | **eGFR < threshold** | **eGFR ≥ threshold** | **eGFR < threshold** | **eGFR ≥ threshold** | **eGFR < threshold** | **eGFR ≥ threshold** |
| <40 | 86 | 3 (12%) | 23 (88%) | 4 (5%) | 72 (95%) | 0 (0%) | 16 (100%) |
| 40-49 | 77 | 4 (7%) | 57 (93%) | 7 (6%) | 104 (94%) | 1 (3%) | 30 (97%) |
| 50-59 | 68 | 5 (5%) | 104 (95%) | 2 (2%) | 132 (98%) | 0 (0%) | 71 (100%) |
| 60-69 | 59 | 0 (0%) | 49 (100%) | 0 (0%) | 114 (100%) | 1 (3%) | 36 (97%) |
| >69 | 50 | 0 (0%) | 5 (100%) | 0 (0%) | 31 (100%) | 0 (0%) | 5 (100%) |
| **Total** | | **12 (5%)** | **238 (95%)** | **13 (3%)** | **453 (97%)** | **2 (1%)** | **158 (99%)** |
| Abbreviations: eGFR: estimated glomerular filtration rate. | | | | | | | |
