# Supplementary material for: Impact of measured versus estimated glomerular filtration rate-based screening on living kidney donor characteristics: A study of multiple cohorts
Source: PLoS One. 2022 Jul 7;17(7):e0270827. doi: 10.1371/journal.pone.0270827 (PMC9262218; doi:10.1371/journal.pone.0270827)
Supplement: S1 File — (DOCX) [file pone.0270827.s012.docx]

**S1 Methods. GFR measurement mGFR-cohort**

GFR was calculated from measurements of the clearance of radiolabeled iothalamate (^125^I-iothalamate).^1^ Before constant infusion of iothalamate started, a blood sample was drawn from the donors. This blood sample was used for routine laboratory measurements. Subsequently, infusion of iothalamate at 0.04 ml/kg body weight was started. The infusion solution contained 0.04 MBq of ^125^I-iothalamate (following an initial dose of 0.6 MBq ^125^I-iothalamate) and 0.03 MBq ^131^I-hippurate and was started at 8:00 a.m. at an infusion rate of 12 ml/h. After a stabilization period, measurements started at 10:00 a.m. Clearances were calculated as (U*V)/P and (I*V)/P, where U*V represents the urinary excretion, I*V represents the infusion rate of the tracer and P represents the plasma tracer concentration per clearance period. From clearance levels of these traces, GFR, effective renal plasma flow, and filtration fraction were calculated. Correction for incomplete bladder emptying and dead space was achieved by multiplying the urinary ^125^I-Iothalamate clearances with plasma and urinary ^131^I-hippurate clearance. The day-to-day variability of the mGFR is 2.5%.^2^

1. Tent H, Rook M, Stevens LA, van Son WJ, van Pelt LJ, Hofker HS, et al. Renal function equations before and after living kidney donation: a within-individual comparison of performance at different levels of renal function. Clin J Am Soc Nephrol. 2010 Nov;5(11):1960–8.

2. Apperloo AJ, de Zeeuw D, Donker AJ, de Jong PE. Precision of glomerular filtration rate determinations for long-term slope calculations is improved by simultaneous infusion of 125I-iothalamate and 131I-hippuran. J Am Soc Nephrol. 1996;7(4):567–72.
