# Supplementary material for: Impact of measured versus estimated glomerular filtration rate-based screening on living kidney donor characteristics: A study of multiple cohorts
Source: PLoS One. 2022 Jul 7;17(7):e0270827. doi: 10.1371/journal.pone.0270827 (PMC9262218; doi:10.1371/journal.pone.0270827)
Supplement: Attachment — Submitted filename: Response to Reviewers.docx [file pone.0270827.s013.docx]

**Reviewers' comments:**

**Have the authors made all data underlying the findings in their manuscript fully available?

Reviewer #1: No**

Author reply: Thank you for reviewing our revised manuscript. We now included a data availability statement at the end of the manuscript.

**Review Comments to the Author

Reviewer #1: Thank you for addressing my concerns, and making significant changes to the analysis and manuscript. The paper is much improved, and the more cautious conclusions more appropriate given the limitations of the study.

Table 2 and Table 3 'P vs. mGFRcohort1' should this be 'vs mGFR-cohort' as there's only one.
Table 2 and Table 4: Length should be height**

Author reply: We changed “mGFR-cohort1” to “mGFR-cohort” and “Length” to “Height” throughout the manuscript and supplementary data accordingly.
